# Supplementary material for: Virtual reality offerings for wellbeing for and by marginalized populations: A scoping review on equity and intersectionality
Source: Glob Ment Health (Camb). 2025 Oct 29;12:e131. doi: 10.1017/gmh.2025.10084 (PMC12641318; doi:10.1017/gmh.2025.10084)
Supplement: Seon et al. supplementary material 1 — Seon et al. supplementary material [file S2054425125100848sup001.docx]

## Medline search strategy

1 (ethnic disparities or ethnic disparity or health disparities or health disparity).ti,ab,cl,oa,kw,kf. or exp healthcare disparities/ or health care disparities.ti,ab,cl,oa,kw,kf. or healthcare disparities.ti,ab,cl,oa,kw,kf. or health-care disparities.ti,ab,cl,oa,kw,kf. or health care disparity.ti,ab,cl,oa,kw,kf. or healthcare disparity.ti,ab,cl,oa,kw,kf. or health-care disparity.ti,ab,cl,oa,kw,kf. or exp health status disparities/ or "disparities in health".ti,ab,cl,oa,kw,kf. or (exp culturally competent care/ or "culturally competent care".ti,ab,cl,oa,kw,kf. or disparities.ti,ab,cl,oa,kw,kf. or health inequality.ti,ab,cl,oa,kw,kf. or health inequalities.ti,ab,cl,oa,kw,kf. or health inequities.ti,ab,cl,oa,kw,kf. or health inequity.ti,ab,cl,oa,kw,kf. or "health related quality of life".ti,ab,cl,oa,kw,kf. or "health-related quality of life".ti,ab,cl,oa,kw,kf. or exp health services accessibility/ or exp health services, indigenous/ or exp health status disparities/ or exp social class/ or social class.ti,ab,cl,oa,kw,kf. or exp social determinants of health/ or "social determinants of health".ti,ab,cl,oa,kw,kf. or social disparities.ti,ab,cl,oa,kw,kf. or social disparity.ti,ab,cl,oa,kw,kf. or social factors.ti,ab,cl,oa,kw,kf. or social inequities.ti,ab,cl,oa,kw,kf. or social inequity.ti,ab,cl,oa,kw,kf. or socioeconomic factor.ti,ab,cl,oa,kw,kf. or exp socioeconomic factors/ or socioeconomic factors.ti,ab,cl,oa,kw,kf. or socioeconomically disadvantaged.ti,ab,cl,oa,kw,kf.)

2 (African American or African Americans or African ancestry).ti,ab,cl,oa,kw,kf. or exp african continental ancestry group/ or Afro-descendent*.ti,ab,cl,oa,kw,kf. or Afro-Caribbean.ti,ab,cl,oa,kw,kf. or Diaspora.ti,ab,cl,oa,kw,kf. or exp ageism/ or AIAN.ti,ab,cl,oa,kw,kf. or Alaska Native.ti,ab,cl,oa,kw,kf. or Alaska Natives.ti,ab,cl,oa,kw,kf. or exp american native continental ancestry group/ or exp apartheid/ or Asian.ti,ab,cl,oa,kw,kf. or exp asian continental ancestry group/ or Asians.ti,ab,cl,oa,kw,kf. or Black American.ti,ab,cl,oa,kw,kf. or Black Americans.ti,ab,cl,oa,kw,kf. or disabled.ti,ab,cl,oa,kw,kf. or exp disabled persons/ or disabled persons.ti,ab,cl,oa,kw,kf. or Invisible disability.ti,ab,cl,oa,kw,kf. or mental health disabilit*.ti,ab,cl,oa,kw,kf. or mental health impairment*.ti,ab,cl,oa,kw,kf. or blindness.ti,ab,cl,oa,kw,kf. or visual impair*.ti,ab,cl,oa,kw,kf. or deaf*.ti,ab,cl,oa,kw,kf. or Neurodiver*.ti,ab,cl,oa,kw,kf. or developmental disability*.ti,ab,cl,oa,kw,kf. or functional limitation.ti,ab,cl,oa,kw,kf. or participation limitation.ti,ab,cl,oa,kw,kf. or diverse population.ti,ab,cl,oa,kw,kf. or diverse populations.ti,ab,cl,oa,kw,kf. or exp "emigrants and immigrants"/ or asylum seeker.ti,ab,cl,oa,kw,kf. or ethnic group.ti,ab,cl,oa,kw,kf. or exp ethnic groups/ or ethnic groups.ti,ab,cl,oa,kw,kf. or ethnic inequalities.ti,ab,cl,oa,kw,kf. or ethnic population.ti,ab,cl,oa,kw,kf. or ethnic populations.ti,ab,cl,oa,kw,kf.

3 (ghetto or ghettos).ti,ab,cl,oa,kw,kf. or exp health services for persons with disabilities/ or Hispanic.ti,ab,cl,oa,kw,kf. or exp hispanic americans/ or Hispanics.ti,ab,cl,oa,kw,kf. or homeless.ti,ab,cl,oa,kw,kf. or exp homeless persons/ or immigrant.ti,ab,cl,oa,kw,kf. or immigrants.ti,ab,cl,oa,kw,kf. or Indian.ti,ab,cl,oa,kw,kf. or Indians.ti,ab,cl,oa,kw,kf. or exp indians, north american/ or Latina.ti,ab,cl,oa,kw,kf. or Latinas.ti,ab,cl,oa,kw,kf. or Latino.ti,ab,cl,oa,kw,kf. or Latinos.ti,ab,cl,oa,kw,kf. or Latinx.ti,ab,cl,oa,kw,kf. or exp mexican americans/ or exp medically underserved area/ or exp medically uninsured/ or minorities' health.ti,ab,cl,oa,kw,kf. or minority group.ti,ab,cl,oa,kw,kf. or exp minority groups/ or minority groups.ti,ab,cl,oa,kw,kf. or exp minority health/ or minority health.ti,ab,cl,oa,kw,kf. or minority population.ti,ab,cl,oa,kw,kf. or minority populations.ti,ab,cl,oa,kw,kf. or migrant worker.ti,ab,cl,oa,kw,kf. or migrant workers.ti,ab,cl,oa,kw,kf. or Native American.ti,ab,cl,oa,kw,kf. or Native Americans.ti,ab,cl,oa,kw,kf. or Native Hawaiian.ti,ab,cl,oa,kw,kf. or Native Hawaiians.ti,ab,cl,oa,kw,kf. or exp oceanic ancestry group/ or Pacific Islander.ti,ab,cl,oa,kw,kf. or Pacific Islanders.ti,ab,cl,oa,kw,kf. or "people of color".ti,ab,cl,oa,kw,kf. or exp poverty/ or poverty.ti,ab,cl,oa,kw,kf. or exp poverty areas/ or "poverty area".ti,ab,cl,oa,kw,kf. or "poverty areas".ti,ab,cl,oa,kw,kf. or exp race factors/ or "race factors".ti,ab,cl,oa,kw,kf. or "race and ethnicity".ti,ab,cl,oa,kw,kf. or "racial and ethnic minorities".ti,ab,cl,oa,kw,kf. or racial discrimination.ti,ab,cl,oa,kw,kf. or racial disparities.ti,ab,cl,oa,kw,kf. or racial disparity.ti,ab,cl,oa,kw,kf. or racial equality.ti,ab,cl,oa,kw,kf. or racial equity.ti,ab,cl,oa,kw,kf. or racial inequities.ti,ab,cl,oa,kw,kf. or racial inequity.ti,ab,cl,oa,kw,kf. or racial prejudice.ti,ab,cl,oa,kw,kf. or racial segregation.ti,ab,cl,oa,kw,kf. or exp racism/ or exp refugees/ or refugees.ti,ab,cl,oa,kw,kf. or exp rural health/ or rural health.ti,ab,cl,oa,kw,kf. or exp rural health services/ or exp rural population/ or rural population.ti,ab,cl,oa,kw,kf. or rural populations.ti,ab,cl,oa,kw,kf. or exp sexism/

4 (slum or slums).ti,ab,cl,oa,kw,kf. or exp social discrimination/ or exp social marginalization/ or exp social segregation/ or "exp transients and migrants"/ or underserved.ti,ab,cl,oa,kw,kf. or exp undocumented immigrants/ or exp medically uninsured/ or uninsured.ti,ab,cl,oa,kw,kf. or vulnerable population.ti,ab,cl,oa,kw,kf. or exp vulnerable populations/ or vulnerable populations.ti,ab,cl,oa,kw,kf. or exp working poor/ or working poor.ti,ab,cl,oa,kw,kf. or bisexuals.ti,ab,cl,oa,kw,kf. or bisexual.ti,ab,cl,oa,kw,kf. or bigender.ti,ab,cl,oa,kw,kf. or female homosexuality.ti,ab,cl,oa,kw,kf. or gay.ti,ab,cl,oa,kw,kf. or gays.ti,ab,cl,oa,kw,kf. or gender change.ti,ab,cl,oa,kw,kf. or gender confirmation.ti,ab,cl,oa,kw,kf. or gender disorder.ti,ab,cl,oa,kw,kf. or gender disorders.ti,ab,cl,oa,kw,kf. or gender dysphoria.ti,ab,cl,oa,kw,kf. or gender diverse.ti,ab,cl,oa,kw,kf. or gender-diverse.ti,ab,cl,oa,kw,kf. or gender diversity.ti,ab,cl,oa,kw,kf. or exp gender identity/ or gender identity.ti,ab,cl,oa,kw,kf. or gender minorities.ti,ab,cl,oa,kw,kf. or "gender non conforming".ti,ab,cl,oa,kw,kf. or gender non-conforming.ti,ab,cl,oa,kw,kf. or gender orientation.ti,ab,cl,oa,kw,kf. or genderqueer.ti,ab,cl,oa,kw,kf. or Agender.ti,ab,cl,oa,kw,kf. or gender reassignment.ti,ab,cl,oa,kw,kf. or gender surgery.ti,ab,cl,oa,kw,kf. or GLBT.ti,ab,cl,oa,kw,kf. or GLBTQ.ti,ab,cl,oa,kw,kf. or exp health services for transgender persons/ or homophile.ti,ab,cl,oa,kw,kf. or homophilia.ti,ab,cl,oa,kw,kf. or homosexual.ti,ab,cl,oa,kw,kf. or exp homosexuality/ or exp homosexuality, female/ or exp homosexuality, male/ or homosexuals.ti,ab,cl,oa,kw,kf. or intersex.ti,ab,cl,oa,kw,kf. or lesbian.ti,ab,cl,oa,kw,kf. or lesbianism.ti,ab,cl,oa,kw,kf. or lesbians.ti,ab,cl,oa,kw,kf. or 2SLGBTQIA.ti,ab,cl,oa,kw,kf. or LGBBTQ.ti,ab,cl,oa,kw,kf. or LGBT.ti,ab,cl,oa,kw,kf. or LGBTI.ti,ab,cl,oa,kw,kf. or LGBTQ.ti,ab,cl,oa,kw,kf. or LGBTQI.ti,ab,cl,oa,kw,kf. or LGBTQIA.ti,ab,cl,oa,kw,kf. or "men having sex with men".ti,ab,cl,oa,kw,kf. or "men who have sex with men".ti,ab,cl,oa,kw,kf. or "men who have sex with other men".ti,ab,cl,oa,kw,kf. or nonheterosexual.ti,ab,cl,oa,kw,kf. or non-heterosexual.ti,ab,cl,oa,kw,kf. or "non heterosexuals".ti,ab,cl,oa,kw,kf. or nonheterosexuals.ti,ab,cl,oa,kw,kf. or pansexual.ti,ab,cl,oa,kw,kf. or polysexual.ti,ab,cl,oa,kw,kf. or queer.af. or "same sex".ti,ab,cl,oa,kw,kf. or exp "sexual and gender disorders"/ or exp "sexual and gender minorities"/ or sex change.ti,ab,cl,oa,kw,kf. or sex reassignment.ti,ab,cl,oa,kw,kf. or exp sex reassignment procedures/ or exp sex reassignment surgery/ or sex reassignment surgery.ti,ab,cl,oa,kw,kf. or sexual diversity.ti,ab,cl,oa,kw,kf. or sexual minorities.ti,ab,cl,oa,kw,kf. or sexual minority.ti,ab,cl,oa,kw,kf. or sexual orientation.ti,ab,cl,oa,kw,kf. or transgender*.ti,ab,cl,oa,kw,kf. or exp transgender persons/ or Non-binary.ti,ab,cl,oa,kw,kf. or "Non binary".ti,ab,cl,oa,kw,kf. or transsexual*.ti,ab,cl,oa,kw,kf. or transman.ti,ab,cl,oa,kw,kf. or trans men.ti,ab,cl,oa,kw,kf. or Trans masc*.ti,ab,cl,oa,kw,kf. or exp transsexualism/ or transsexualism.ti,ab,cl,oa,kw,kf. or transwoman.ti,ab,cl,oa,kw,kf. or trans women.ti,ab,cl,oa,kw,kf. or transwomen.ti,ab,cl,oa,kw,kf. or Trans femme.ti,ab,cl,oa,kw,kf. or "two spirit".ti,ab,cl,oa,kw,kf. or two-spirit.ti,ab,cl,oa,kw,kf. or "women who have sex with women".ti,ab,cl,oa,kw,kf.

5 exp Indigenous people/ or (Aborigin* or Amerind* or autocht* or Eskimo* or indigenous or Indigena* or Inuit* or Innuit* or Inuk or Inupiat* or "First Nation" or "First Nations" or "First People*" or Kalaallit* or Metis or Native or Natives or Mestizo or "Native Hawaiian*" or "pacific islander*" or "torres strait islander*" or Inupiaq or yupik or "yup'ik" or saami or samis or maori* or maoori* or Bedouin*).ti,ab,cl,oa,kw,kf. or ((indigenous or native or "first peoples" or India* or Bangladesh* or Bhutan* or Nepal or Pakistan* or Sri Lanka* or Greenland* or Africa* or Australia* or "Torres Strait Island*" or aotearoa* or "new Zealand*" or "central America*" or "south America*" or Mexic* or Palestin* or "Middle East*" or Russia* or Caribbean*) and (circumpolar or tribe or tribes or tribal or clan or clans or ("traditional medicine" not Chinese) or shaman* or shamanism or "country food" or reservation or nomad*)).ti,ab,cl,oa,kw,kf.

6 "VR".ti,ab,cl,oa,kw,kf. or (("3-dimensional" or "three-dimensional" or "3-D") adj ("scene" or "game" or "treatment" or "intervention" or "place" or "avatar" or "immersion" or "space" or "semi-immersive" or "immersive" or "non-immersive" or "CBT" or "cognitive-behavioural therapy" or "exposure therapy" or "environment" or "video" or "film" or "training")).mp. or (virtual adj ("scene" or "game" or "treatment" or "intervention" or "place" or "avatar" or "immersion" or "space" or "semi-immersive" or "immersive" or "non-immersive" or "CBT" or "cognitive-behavioural therapy" or "exposure therapy" or "environment" or "video" or "film" or "training")).mp. or exp Virtual Reality/ or exp Augmented Reality/ or ("virtual reality" or "augmented reality" or "merged reality" or "mixed reality" or "extended reality" or "virtual training").mp. or "virtual environment".mp. or ("vr-cbt" or "virtual reality cognitive behavioural therapy" or "vret" or "virtual reality exposure therapy" or "virtual reality assisted cognitive behavioural therapy" or "cbt-vr").mp. or ("CAVE" or "haptic" or "360 degree video" or "360 video" or "360 VR" or "desktop VR").mp. or virtual training.mp. or Virtual Reality Exposure Therapy/ or avatar.mp.

7 1 or 2 or 3 or 4 or 5

8 6 and 7

**Resources for marginalized individuals and researchers**

The following resources and programs were identified during the current review. These programs were used, studied or developed by the researchers and are available for use by the public. The programs may be free, have paid features, or require a subscription. Each resource is listed by its name, researchers or company who developed the application, details, and URL.

| **Program** | **Owner** | **Description** | **Link** |
| --- | --- | --- | --- |
| VRChat | VRChat Inc | This social VR platform has various groups, such as G-A-Y Talk, G-A-Y Meet-up social, LGBTQ+ and Friends Meetups and Hangouts as seen in Acena and Freeman, 2021; Freeman and Acena, 2022; G. Freeman et al. 2022; Li et al. 2023. | <https://hello.vrchat.com/> |
| gameChange VR | BehaVR, RealizedCare | Seen in Altunkaya et al. 2022; Bond et al. 2023; D. Freeman et al. 2022; D. Freeman et al. 2022 the program was used in populations with severe mental illness. | <https://www.realizedcare.com/agoraphobic-avoidance> |
| Wii | Nintendo | Researchers used Wii Fit and Wii Sports in Kwon et al. 2022; Singh et al. 2017 to support adults with disabilities. | <https://www.nintendo.com/en-ca/> |
| Oculus Medium | Meta | Paré et al. 2019 used this drawing and 3D art program. | <https://www.meta.com/experiences/pcvr/1336762299669605/> |
| Facebook Spaces | Meta | Paré et al. 2019 used this social VR platform. | <https://horizon.meta.com/> |
| Floreo VR | Floreo | Wang et al. 2023 used this skills-based program for social competency and social support | <https://floreovr.com/> |
| Google Arts and Culture | Google | The platform offers multiple arts and culture tours in augmented reality, as seen in Wang et al. 2023 combined with their own curriculum. | <https://artsandculture.google.com/> |
| Evenness | Devika Group | A sensory room as seen in Mills et al. 2023 | <https://www.evenness.app/experiences-art-therapy> |
| Guided Meditation VR | Meta | A sample of homeless youth reported on their experiences with this meditation program in Chavez et al. 2020. | <https://www.meta.com/experiences/guided-meditation-vr/929143807179080/> |
| Second Life | Linden Research, Inc | Used by Shah et al. 2015; Nosek et al. 2016; Nosek et al. 2019; Robinson-Whelen et al. 2020; Mitchell et al. 2022; Mitchell et al. 2023; Rosal et al. 2014; Johnson et al. 2014; Bloustien and Wood, 2015; Davis and Chansiri, 2019; Stendal and Balandin, 2015; Abal, 2012 for creating private and customized virtual environments. None of these to our knowledge are available to the public, except those when public groups were being studied. | <https://secondlife.com/> |
