# Supplementary material for: Virtual reality offerings for wellbeing for and by marginalized populations: A scoping review on equity and intersectionality
Source: Glob Ment Health (Camb). 2025 Oct 29;12:e131. doi: 10.1017/gmh.2025.10084 (PMC12641318; doi:10.1017/gmh.2025.10084)
Supplement: Seon et al. supplementary material 2 — Seon et al. supplementary material [file S2054425125100848sup002.pdf]

Strengthening the Integration of Intersectionality Theory in Health Inequality Analysis (SIITHIA) checklist

How to cite this document

Public Health Agency of Canada. How to integrate intersectionality theory in quantitative health equity analysis? A rapid review and checklist of promising practices. Ottawa, ON: PHAC; 2022.

Study/ Report section	Item	Promising practice	ü
**Introduction**			
Background/ Rationale	1.	Provide a well-referenced definition of intersectionality theory, which alludes to its central principles*	X
	2.	Describe inequalities that are consistently observed between population groups, and that are assumed to be avoidable, as "unjust/unfair" and requiring action.	X
	3.	Describe the known determinants of the outcome of interest that operate at, and above, the individual level.	X
	4.	State and describe underlying assumptions underpinning the study, including a reflexivity* or positionality* statement from the research team.	X
	5.	Integrate and summarize evidence developed through research and analysis that involve populations that are affected by the inequalities under study or forms of knowledge that have been under-represented in public health practice.	X
Objectives	6.	Draw on, and describe literature and complementary theoretical frameworks (including those from outside the field of health sciences), as needed, to justify and frame the research questions and objectives.	X
	7.	Explore one or more objectives relevant to intersectionality theory, including:	
		a) Assessing effect modification or interaction between two or more measures capturing axes of marginalization, in determining health and social outcomes and inequalities.	X
		b) Exploring to what extent observed health and social inequalities are explained by a given sub-set of characteristics or factors at the individual, community, or societal level.	
		c) Assessing changes in determinants, outcomes and inequalities therein (i.e., associations between determinants and outcomes), over time and across contexts.	
	8.	Engage with people and populations that are affected by the inequalities under study when establishing research questions and objectives.	X
**Methods**			
	9.	Engage with populations that are affected by the inequalities under study, when designing the methods.	X
Data source(s)		Where possible and relevant to the research question:	
	10.	Collect or use data that allow a comparison of outcomes across intersecting social positions* .	X
	11.	Collect or use data that allow for an assessment of heterogeneity in determinants and outcomes* across social or spatial units of aggregation (e.g., schools, regions).	X

	12.	Collect or use data that allow for an assessment of heterogeneity in outcomes across time (including temporal contexts based on calendar time, and inter-generational and lifecourse perspectives).	X
	13.	Collect or use data that allows for an assessment of independent measures that are hypothetically modifiable, and therefore amenable to intervention.	N/A
	14.	Collect or use qualitative data (e.g., using interviews, focus groups, open-ended survey questions, program evaluations, etc.) to complement quantitative data sources, in a mixed-methods research design approach.	X
Measures		Where relevant to the research question, operationalize independent measures that enable an assessment of outcomes across:	na
	15.	Two or more axes of marginalization;	
	16.	Units of social or spatial aggregation or clustering;	
	17.	Temporal contexts (including contexts based on calendar time, and inter-generational and lifecourse perspectives).	
	18.	Operationalize and utilize independent measures that are hypothetically modifiable, and therefore amenable to intervention.	
	19.	Describe assumptions about the relationship between study measures, including the assumed direction and temporal ordering of associations, using a causal map or Directed Acyclic Graph* .	
	20.	Describe assumptions about the broader social phenomena that measures are assumed to capture or represent.	
	21.	Describe and justify selected reference categories.	
Analysis	22.	Select/design analyses according to study objectives. For example:	
		a) For objective 7a (assessing effect modification between two or more measures) potential analyses could include i) stratified analyses, ii) regression-based analyses with interaction terms, iii) or multilevel analysis of individual heterogeneity and discriminatory accuracy (MAIHDA) analyses	X
		b) For objective 7b (exploring determinants of inequalities), potential analyses could include i) causal mediation or ii) decomposition analyses	
		c) For objective 7c (assessing changes across time and place), potential analyses could include i) stratified descriptive analyses based on social, temporal, and/or geographic contexts, ii) multivariate mixed-effects regression analyses	
	23.	Assess both absolute and relative inequalities between groups.	na
	24.	In regression-based analyses, use a parsimonious set of adjustment variables based on the causal map described (Item #19).	na
	25.	State and test underlying analytic assumptions using sensitivity analyses.	na
	26.	Where relevant to the research question, analyze qualitative data, using methods most appropriate for the study's objectives.	X
**Results**			
	27.	Present and discuss determinants, outcomes, and inequalities therein, stratified by i) relevant sub-groups, ii) units of space, iii) units of time.	X
	28.	Present and interpret effect modification results, distinguishing between additive and multiplicative interaction.	na
	29.	If available and relevant for the research question, present the results of the mixed-methods or qualitative analyses that were performed (Item #26), including key illustrative quotations.	na

Discussion			
	30.	Engage with populations that are affected by the inequalities under study, for the review and interpretation of findings.	X
Findings	31.	Refer to principles of intersectionality theory when interpreting the plausible mechanisms explaining results.	n a
Implications	32.	Describe the implications of the study for public health practice, as well as policy and systems change.	X
	33.	Describe the implications of the study for the potential population targets of intervention (e.g., universal policy, targeted/proportional universalist policy).	na
Limitations	34.	Describe how key/core principles of intersectionality were or were not integrated in the study.	X
	35.	Describe any limitations of data sources (including statistical power), measures and analyses, and their implications.	X
	36.	Include reflexivity about the power invested in (and reproduced by) the methods used.	X
* Please refer to the report's Glossary section for definitions			

Updated: June 10, 2022.
